# Reliability, validity, interpretability and responsiveness of the DEMMI mobility index for Brazilian older hospitalized patients

**DOI:** 10.1371/journal.pone.0230047

**Published:** 2020-03-18

**Authors:** Lucas Spadoni Tavares, Nayara Alexia Moreno, Bruno Garcia de Aquino, Larissa Francielly Costa, Ivens Willians Silva Giacomassi, Maria do Socorro Morais Pereira Simões, Adriana Cláudia Lunardi

**Affiliations:** 1 Master and Doctoral Programs in Physical Therapy, Universidade Cidade de São Paulo, São Paulo, Brazil; 2 Division of Physical Therapy, Instituto de Assistência Médica ao Servidor Público Estadual, São Paulo, Brazil; 3 Department of Human Movement Sciences, Universidade Federal de São Paulo, Santos, Brazil; 4 Departament of Physical Therapy, Faculdade de Medicina da Universidade de São Paulo, São Paulo, Brazil; eCampus University, ITALY

## Abstract

**Aim:**

To translate and adapt cross-culturally the *De Morton Mobility Index* from English to Brazilian Portuguese. Furthermore, to test the content validity, reliability, construct validity, interpretability and responsiveness for older hospitalized patients.

**Methods:**

After we carried out the translation and the cross-cultural adaptation of the *De Morton Mobility Index* and its administration instructions according to international guidelines, the content validity of *De Morton Mobility Index* was tested by experienced physiotherapists. In the sequence, the reliability, construct validity, interpretability and responsiveness were tested in a test-retest design with 93 older patients hospitalized in ward for clinical reasons. The reliability was tested by Cronbach's alpha coefficient (internal consistency), standard error measurement (agreement), and interclass correlation coefficients (intra and inter-examiner reliability). The construct validity was tested by Pearson's correlation between the *De Morton Mobility Index* score and the number of steps. Interpretability was analyzed by determining the minimum detectable change and the floor and ceiling effects (frequency of maximum and minimum scoring). Responsiveness was analyzed by effect size.

**Results:**

The Brazilian version of the *De Morton Mobility Index* was made and adapted. The internal consistency (α = 0.89), reliability intra-(ICC = 0.94) and inter-examiners (ICC = 0.82), agreement were all adequate. The *De Morton Mobility Index* is validity when correlated with number of steps (r = 0.46). Floor or ceiling effects (<15%) were not observed and the responsiveness was high (ES = 3.65).

**Conclusion:**

The *De Morton Mobility Index* has shown adequate reliability, validity, interpretability and responsiveness for the evaluation of the mobility of older hospitalized patients.

## Introduction

Reduction of mobility is a major cause of the lower quality of life and limited social participation[1–3]. In particular, reduction of mobility is commonly seen in older hospitalized patients[4] and results in an increased risk of falls, longer hospital admissions, more severe disability and morbidity, and higher mortality rates[5–7]. To manage older patients’ mobility function, a reliable and valid measure assessing mobility is a prerequisite[8].

The mobility of older patients tends to be evaluated by performance-based assessments[9–11]. Specifically, the Timed Up and Go and the Six-Minute Walk Test are two commonly used measures particularly in older hospitalized patients. However, previous studies showed that these measures have ceiling effects in older hospitalized patients, which severely limit their ability to measure older patients’ mobility function[12]. Thus, the commonly used measures cannot validly assess older patients’ mobility.

An instrument specifically developed and validated with this goal was the *De Morton Mobility Index* (DEMMI), developed and validated specifically for older patients hospitalized in ward[8,13]. The DEMMI evaluates 15 activities divided into 5 groups: in-bed activities, on the chair, static balance, ambulation and dynamic balance. Scoring is based on the patient’s performance in each of the activities and on the level of assistance needed for their execution[8,13].

The DEMMI was developed in English and previously translated into different languages[14–16]. Most language versions of the DEMMI were translated through a rigorous procedure for cross-cultural validation and adaption[17]. Moreover, good psychometric properties have been shown in previous studies in most language versions, supporting that the DEMMI is a promising measure to assess older patients’ mobility function[18]. However, the DEMMI has no Brazilian Portuguese version, limiting its utility. Thus, the aim of this study has been to translate and adapt cross-culturally the DEMMI from English to Brazilian Portuguese. Moreover, the psychometric reliability, validity, interpretability, and responsiveness of the DEMMI were validated in older hospitalized patients.

## Methods

### Design

To translate and adapt cross-culturally the *De Morton Mobility Index* from English to Brazilian Portuguese. Furthermore, to test the content validity, reliability, construct validity, interpretability and responsiveness for older hospitalized patients.

### Participants

At the pre-testing stage, this study involved 7 physiotherapists with at least 5 years’ experience in caring for older patients in hospitals. For the properties of measurement test stage, following COSMIN[18] guidelines, 100 older patients (60 years old or more) hospitalized for clinical reasons in ward at University hospital, not prescribed with restriction to bed and capable of understanding the instructions of examiners were included. Patients who had shown altered clinical condition or who had been discharged from the hospital between the test and retest were excluded. This project was approved by the Ethics Committees from University and from Hospital. All participants signed a Term of Free and Informed Consent.

### Proceedings

Aiming to apply the DEMMI in our population, a Brazilian Portuguese version must be made. After the translation and cross-cultural adaptation (translation, back translation, experts’ committee, pretest and final version) from English to Brazilian Portuguese following international guidelines by two independents and bilingual persons in all stages[17], the instructions for application of the DEMMI were presented to the physiotherapists. The physiotherapists enrolled in pre-testing stage received instructions to apply the DEMMI to older individuals during their work routine and point out the difficulties in the use of the instrument. Aiming to test the content validity, the physiotherapists reported the difficulties in the interpretation of the items of the DEMMI and its pre-test in older hospitalized individuals[18]. All comments from the physiotherapists were considered and the Brazilian Portuguese version of the DEMMI was adjusted by the researchers. After that, the properties of measurement were tested on the patients.

Age, sex, body mass index and cause of hospitalization were recorded in the baseline. The DEMMI was applied (test) by examiner A. After 1 hour, the DEMMI was applied by examiner B (testing reliability inter examiners) and the accelerometer was applied to the patient (test of construct validity). After 24 hours, the retest of the DEMMI was applied by examiner A (testing reliability intra examiners) and the accelerometer was taken off. At the date of hospital discharge, the DEMMI was applied again (test of responsiveness) not necessarily by the same initial examiner.

### Evaluations

#### Mobility

Evaluated via DEMMI[8]. The classification of mobility is based on professional observation of each activity with the following options: incapacity of performing, capacity of performing with help or independence_._ Scoring varies from zero to 19 points. A conversion table allows for the transformation of the raw score into a specific score, called *DEMMI score*, which varies from 0 to 100 points, with higher scores indicating higher levels of mobility[13].

#### Accelerometry

Evaluated the level of physical activity using Actigraph GT3X (Actigraph Corp., USA), installed on the dominant side of the patient’s wrist according to the patient’s report[19]. The device was calibrated during 24 hours between the examiner’s test and retest. The accelerometer was waterproof and could also be used during baths. The time percentage variables in different intensities of activity were used to characterize the sample[20]. The number of steps was recorded[19].

### Test of properties of measurement

Reliability (internal consistency, agreement, intra- and inter-examiner reliability), validity, interpretability (minimum detectable change and ceiling and floor effects) and responsiveness were tested in a test and retest model[21].

#### Internal consistency

It’s the property connected with the relation between the instrument’s items. The internal consistency was evaluated by Cronbach's alpha coefficient (α). Internal consistency was considered adequate if α was between 0.70 and 0.95[22]. Above 0.95 the instrument is considered redundant, that is, more than one item evaluates the same result.

#### Agreement

It’s the property related to the absolute error of the measurement taken by the instrument, that is, there is agreement when two or more measurements repeated in the same clinical condition are similar[23]. Agreement was tested by the standard error of measurement (SEM) between the test and retest was calculated using the formula SEM  = SD_difference_ /√2, where SD_difference_[24], considering the SD = standard deviation. The classification adopted was: SEM <5% of total score = very good, from ≥5% to <10% = good, from ≥10% to <20% = doubtful, >20% = unreliable[22].

Intra and inter-examiner reliability: It’s the property related to how much the instrument is free from measurement errors. Reliability was tested by the interclass correlation coefficient (ICC), subtype absolute agreement for single measurements. The variance of the measures was considered for each individual and not in the group’s average (ICC_2,1_), with its respective confidence interval of 95% (CI95%). The classification adopted was: <0.40 = low, from 0.40 to 0.75 = moderate; from 0.76 to 0.90 = substantial and >0.90 = excellent[22,25]. Bland-Altman plots were also built for intra e inter examiners agreements.

#### Construct validity

It is the property that shows if the instrument tested evaluates the construct proposed compared to another instrument that evaluates the same construct[26]. The validity was tested by the Pearson correlation between the score in the DEMMI scale and the number of steps gaged by accelerometry. The classification adopted was: *r* <0.30 = weak, from 0.30 to 0.60 = moderate and >0.60 = strong[18]. The *a priori* hypothesis was that the correlation between the DEMMI and accelerometry was positive (concerning direction) and moderate (regarding the magnitude) (≥0.30 r <0.60).

#### Interpretability

It is the property dealing with the internal error of the instrument, that is, what is the minimum variation that when detected indicates a clinical change and not a measurement error inherent to the instrument[24]. Interpretability was analyzed through the calculation of the minimum detectable change with 90% confidence (MDC_90_), and determination of the floor and ceiling effects. MDC_90_ was calculated as follows: MDC_90_ = score in the test, subtracted from the score in the retest, divided by √2×SEMx1.64[27], and the floor and ceiling effects were considered present if 15% or more of the individuals reached the minimum or maximum score in the evaluation[22].

#### Responsiveness

It is the capacity of the questionnaire to identify possible changes in the construct associated with the clinical condition over time[18]. This responsiveness was measured by the effect size (ES)[28]. ES was calculated by the variation of the score in the DEMMI at the moment of discharge from the hospital in relation to the score in the test, divided by the standard deviation of the score in the test[29]. The classification adopted was the propose by Cohen: ES ≤0.20 = small, from 0.21 to 0.50 = moderate and ≥0.80 = large[30].

## Results

After the translation and cross-cultural adaptation, the Brazilian Portuguese version of the DEMMI was sent to 10 physiotherapists, of whom seven (8.8±4.2 years of experience in hospitals) agreed to participate in the pre-test. Five physiotherapists did not report doubts or problems in the application of the DEMMI. Two physiotherapists reported the following doubts concerning the application of the scale: “What are the 10 seconds mentioned in activity 4? Should the individual be able to remain seated for at least 10 seconds or did he remain only 10 seconds?”, “In activity 11, what does +/- mean? Does it mean with/without?” Each doubt was elucidated. The two physiotherapists agreed with the modifications in the instrument and the final version was confirmed (S1 Fig).

**Fig 1:**
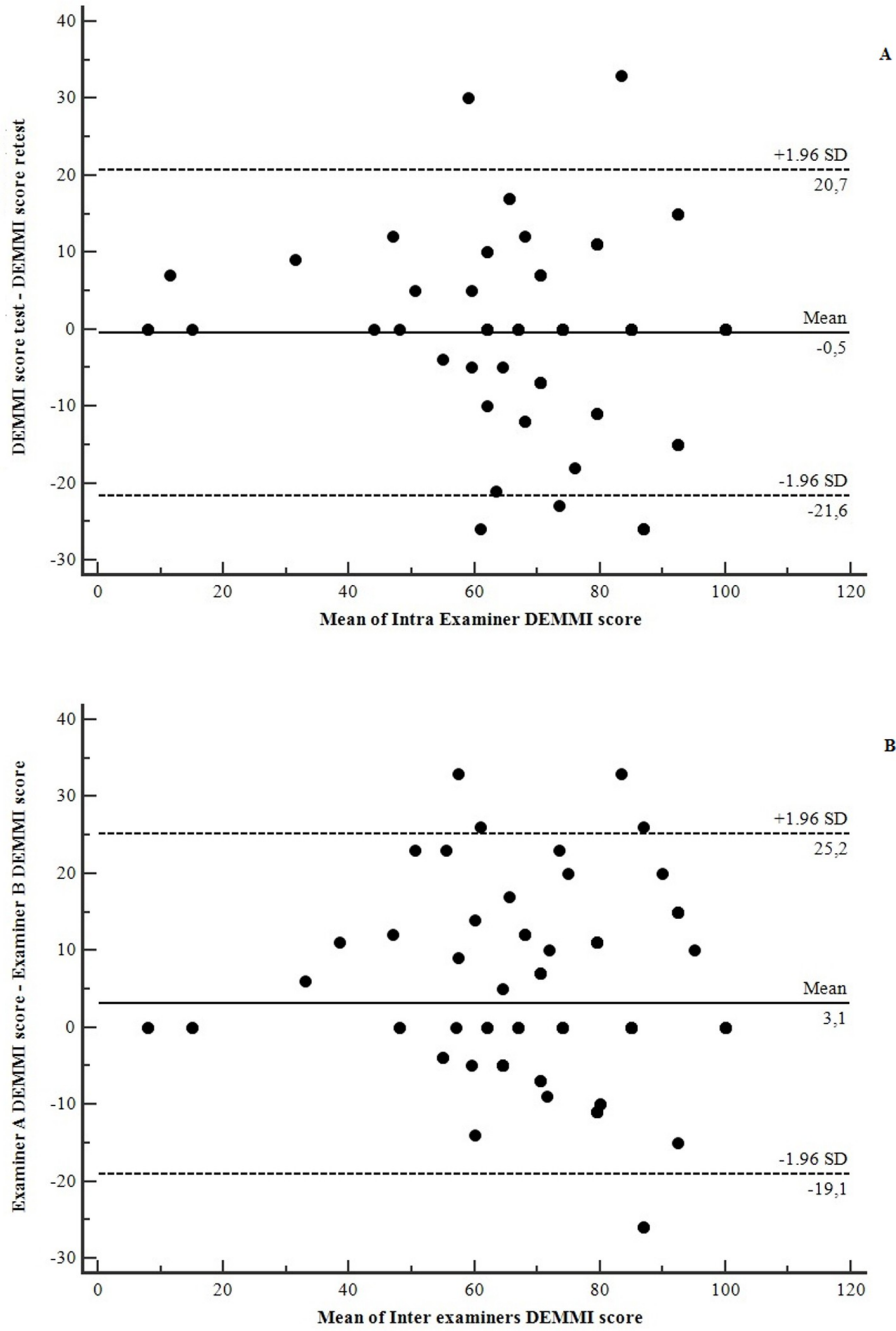
Bland-Altman of agreement for intra examiner (A) and inter Examiners (B). SD = Standard Deviation.

At the stage of the properties of measurement test, 100 older patients consecutively hospitalized were chosen. However, five of them refused to participate in the study, one was discharged from the hospital between the test and retest and one was transferred to the intensive care unit. The average hospitalization time of the patients who finished the study was of 8.1±2.3 days. The characteristics of the individuals are presented in Table 1. The analysis of properties is shown in the Tables 2 and 3 and Fig 1.

**Table 1:** Characteristics of hospitalized older individuals (n = 93).

Variables	Values
Age, years	70.4 ± 8
Male sex	46 (49.5%)
BMI, kg/m^2^	26 ± 6.5
**Cause of hospitalization, n (%)**	
Pneumonia	30 (32.3%)
Exacerbation of chronic pulmonary disease	29 (31.2%)
Neoplasia	16 (17.2%)
Cardiovascular disease	6 (6.4%)
Others	12 (12.9%)
**DEMMI, points**	
Test (examiner A)	72.4 ± 19.5
Test (examiner B)	69.8 ± 20.2
Retest (examiner A)	72.9 ± 20.7
At hospital discharge	72.5 ± 20.2
**Accelerometry, average ± SD**	
% time in sedentary behavior	60.2 ± 10.9
% time in light activity	36.2 ± 9.1
% time in moderate activity	3.6 ± 2.9
Number of steps in 24 hours	8015.3 ± 3927.8

Data presented in average ± standard deviation or absolute number (percentage in the sample); BMI = body mass index; DEMMI = De *Morton Mobility Index*.

**Table 2:** Value of Cronbach's alpha coefficient with exclusion of DEMMI item by item (n = 93)[8].

Activity	Value of Alpha
1. Bridge exercise	0.895
2. Rolling on one side	0.898
3. Lying down to sitting	0.882
4. Sitting without support on chair	0.888
5. Sitting to standing from chair	0.889
6. Sitting to standing without using arms	0.885
7. Standing without support	0.886
8. Standing with feet joined	0.886
9. Standing on tips of feet	0.889
10. Tandem with eyes closed	0.900
11. Distance walked	0.881
12. Independence during walk	0.882
13. Collecting pen from floor	0.886
14. Taking four steps backwards	0.887
15. Jumping	0.905

**Table 3:** Classification of clinimetric properties of DEMMI applied to hospitalized older individuals, according to Mokkink et al[21].

Property	Values	Classification
**Internal consistency**		
Cronbach's alpha coefficient	0.896	Adequate
**Reliability** (ICC_2.1_ (CI95%))		
Intra Examiners	0.92 (0.88 to 0.94)	Excellent
Inter Examiners	0.84 (0.77 to 0.89)	Substantial
**Agreement**		
Standard Error of measurement	0.007%	Very good
**Validity**		
Validity of Construct (r)	0.46	Moderate
**Interpretability**		
Minimum detectable difference	1.83	
Ceiling Effect	13.9%	Appropriate
Floor Effect	0%	Appropriate
**Responsiveness**		
Internal Responsiveness (ES)	3.65	Great

ICC = Interclass correlation coefficient, CI = Confidence interval, r = correlation factor.

## Discussion

Our results show that the Brazilian Portuguese version of the DEMMI has adequate content validity. Besides that, internal consistency, agreement, intra and inter-examiner reliability, construct validity, interpretability and responsiveness were tested for the first time together and following the recommendations of COSMIN[21]. Comparing with other previously published versions, most of the properties that had already been tested presented values similar to those found in this study. Aiming to compare and summarize the properties of measurement tested in the DEMMI versions, the values and mode of analysis between the available versions are presented in Table 4.

**Table 4:** Clinimetric properties of all DEMMI published versions.

Properties	This study	English[8]	German[14]	Dutch[15]	Danish[16]
**Internal consistency**	α = 0.89	NR	α = 0.87	NR	NR
**Reliability**					
**Intra Examiner**	ICC = 0.92 (CI95% 0.88–0.94)	r = 0.94 (CI95% 0.86–0.98)	NR	ICC = 0.94 (CI95% 0.86–0.98)	NR
**Between Examiners**	ICC = 0.84 (CI95% 0.77–0.89)	NR	ICC = 0.94 (CI95% 0.88–0.97)	ICC = 0.85 (CI95% 0.71–0.93)	NR
**Agreement**	0.66 (SEM)	4.10 (SEM)	2.34 (SEM)	2.9 (SEM)	0.78 (SEM)
**Validity**					
**Construct validity**	r = 0.46 (number of steps)	r = 0.68 (Barthel)	r = 0.67 (TUG)	r = 0.73 (TUG)	r = 0.76 (CAS)
**Responsiveness**					
**Internal Responsiveness**	ES = 3.65	ES = 0.39	NR	NR	NR
**Interpretability**					
**Minimum detectable difference**	1.83 (MDD _90%_)	9.43 (MDD _90%_)	8.8 (MDD _90%_)	6.7 (MDD _90%_)	8.16 (DMCI)
**Floor effect**	0%	<1%	NR	0	39%
**Ceiling effect**	13.97%	3.8%	NR	12%	NR

NR = Not reported, α = Cronbach's alpha coefficient, ICC = interclass correlation coefficient, CI = Confidence interval, SEM = Standard error of measurement, MDD = Minimum detectable difference, r = correlation factor, ES = Effect size, HABAM = *Hierarchical Assessment of Balance and Mobility*, TUG = *Time up and go*, CAS = *Cumulated Ambulation Score*, Barthel = *Barthel Index*.

Testing the internal consistency of the Brazilian Portuguese version, the Cronbach's alpha coefficient of the DEMMI found was 0.90. These findings are similar to those in the German version[14], suggesting that the Brazilian Portuguese version can provide a reliable assessment of mobility function in older patients. Moreover, all alpha values were less than 0.90, indicating that no redundant exists in the DEMMI[21]. The values reported by all authors classify agreement as very good in all versions[8,14,15,16]. Accordingly, these findings suggest that the Brazilian Portuguese version of the DEMMI also appears to be a useful measure assessing patients’ mobility function

The test-retest reliability of the Brazilian Portuguese version found that ICC ranged from 0.84 to 0.92, what was similar to observed in the German[14] and Dutch[15]. Only the original English[8] version tested the reliability by the Pearson's correlation (r) between the scores obtained in the test and retest, and was considered strong (r = 0.94). Use of the ICC test is currently recommended by COSMIN[21] and has been followed by the latest versions. The results found for the Brazilian Portuguese version are classified as excellent when the same examiner applies the DEMMI in two different moments for the same patient and as substantial when the DEMMI is applied by different examiners.

In the analysis of validity, the Brazilian Portuguese version presented moderate correlation (r = 0.46) with the number of steps. Other versions that also analyzed this property showed stronger correlations (ranging from 0.67 to 0.76). It is important to stress the differences between the instruments used for comparison. In our study, we used for comparison an objective measurement of the level of physical activity during 24 hours, using accelerometry. The other studies used for comparison the Barthel[8] index, Timed Up and Go test[14,15] and Cumulated Ambulation Score[16]. Among all those analyzed, only the Cumulated Ambulation Score presents domains closer to the construct of the DEMMI, which explains the greater correlation between evaluations. As this instrument is not validated and available in our language, the Barthel index evaluates functional independence through questionnaires, and not by objective quantification of the activity, and TUG demands minimum physical and cognitive conditions for the performance of the test, which takes some seconds[31], we opted for using the number of steps in the comparison with the DEMMI, even if obtaining a lower correlation value. We believe that the number of steps taken in 24 hours reflects adequately how mobile the patient was in that period.

No floor or ceiling effects were observed in Brazilian Portuguese version, probable because our sample was composed by old and very old individuals in different clinical situations, which outlines the potential of this instrument to evaluate patients in different clinical states. The only previous version that reported a floor effect was the Danish[16], however the individuals involved in their study were in their first day after hip fracture corrective surgery. In this kind of surgery, the individual cannot discharge all his weight on the operated lower limb and the pain may be significant at this time[32]. As for the ceiling effect, our results were very close to Danish version, probably because the patients in our sample had their mobility preserved in the first evaluation and took 8000 steps in 24 hours during hospitalization. Maybe in a more debilitated population the frequency of patients with high scores in the DEMMI would be lower.

Our diversified sample may also have caused the lower MDC (1.83) already reported for the DEMMI by the other versions, at less than 2 points. This fact means that any change greater than 2 points in 100 may be considered clinical and not an internal error of the instrument[23]. This stability of the instrument was also observed by the evaluation of agreement, which was considered very good in all versions already produced.

Internal responsiveness presented adequate values (ES = 3.65) for Brazilian Portuguese version. Our result shows that the DEMMI is capable of detecting changes in the level of mobility during the hospitalization period even if our sample has not remained hospitalized for a long period, and even if they have shown an average score of 72 points in 100, showing good mobility, in the first application of the DEMMI.

The major limitation of this study was the sample of 93 individuals. Terwee et al.[22] recommends 100 participants for studies with unidimensional instruments and analysis of reliability. However, samples with 80 participants or more are considered a good size for the required statistical tests[22]. Another limitation was the non-reporting of a priori property hypotheses, besides their validity. This is important to reduce the risk of bias in the studies, but we believe it did not interfere with our results. Finally, we believe there may be a difference in the measurement properties of DEMMI when applied to more debilitated and dependent hospitalized patients than our sample. We recommend that studies in other fragile populations be conducted.

Therefore, we conclude that the DEMMI shows adequate reliability, validity, interpretability and responsiveness for the evaluation of hospitalized older patients. Thus, we recommend the use of this instrument for the evaluation of mobility in the hospital environment, both in practice and in clinical research.

## Supporting information

S1 Fig Final version in Brazilian Portuguese of DEMMI and its instructions to apply. (DOCX)
