# Supplementary figures and images for: Reliability, validity, interpretability and responsiveness of the DEMMI mobility index for Brazilian older hospitalized patients
Source: PLoS One. 2020 Mar 18;15(3):e0230047. doi: 10.1371/journal.pone.0230047 (PMC7080236; doi:10.1371/journal.pone.0230047)

**
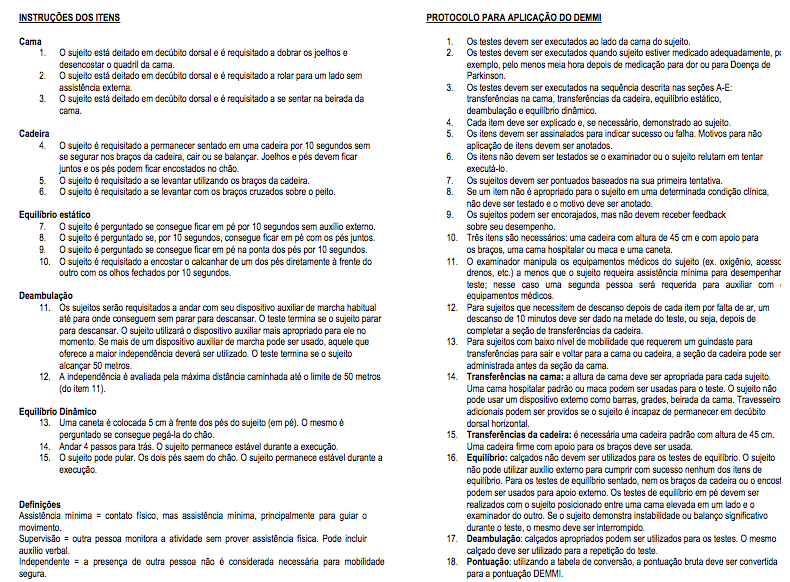
**

Supplement: S1 Fig — (DOCX) [file pone.0230047.s001.docx]
